# Metabonomics Study on Serum Characteristic Metabolites of Psoriasis Vulgaris Patients With Blood-Stasis Syndrome

**DOI:** 10.3389/fphar.2020.558731

**Published:** 2020-11-18

**Authors:** Li Li, Dan-ni Yao, Yue Lu, Jing-wen Deng, Jian-an Wei, Yu-hong Yan, Hao Deng, Ling Han, Chuan-jian Lu

**Affiliations:** ^1^ Molecular Biology and Systems Biology Team of Chinese Medicine, Guangdong Provincial Hospital of Chinese Medicine (The Second Clinical College of Guangzhou University of Chinese Medicine, Guangdong Provincial Academy of Chinese Medical Sciences), Guangzhou, China; ^2^ Department of Dermatology, Guangdong Provincial Hospital of Chinese Medicine (The Second Clinical College of Guangzhou University of Chinese Medicine, Guangdong Provincial Academy of Chinese Medical Sciences), Guangzhou, China

**Keywords:** metabonomics, psoriasis, traditional Chinese medicine syndrome, blood-stasis syndrome, biomarker

## Abstract

Psoriasis is a chronic, refractory, systemic inflammatory skin disease. Traditional Chinese medicine (TCM) shows unique advantage in the treatment of psoriasis based on syndrome differentiation. An untargeted high-throughput metabonomics method based on liquid chromatography coupled to mass spectrometry was applied to study the serum metabolic characteristics in different TCM syndrome types in patients with psoriasis vulgaris (PV), and to discover potential serum biomarkers for its pathogenesis on the endogenous metabolite differentiation basis. The serum metabolic profiles of 45 healthy controls and 124 patients with PV (50 in the blood-stasis group, 30 in the blood-heat group, and 44 in the blood-dryness group) were acquired. The raw spectrometric data were processed using multivariate statistical analysis, and 14 biomarkers related to TCM syndrome differentiation and psoriasis types were screened and identified. The blood-stasis syndrome group showed abnormal lipid metabolism, which was characterized by a low level of phosphatidylcholine (PC) and a high level of lysophosphatidylcholine (LPC). We propose that platelet-activating factor can be applied as a potential biomarker in clinical diagnosis and differentiation of PV with blood-stasis syndrome. The difference in the serum metabolites among PV types with different TCM syndromes and healthy control group illustrated the objective material basis in TCM syndrome differentiation and classification of psoriasis.

## Introduction

Psoriasis is a chronic, refractory, systemic inflammatory skin disease, and it is also quite complex. The etiology and pathogenesis are unclear, and it is challenging to cure (Nestle et al., 2009). In China, long-term clinical practice has proved that traditional Chinese medicine (TCM) syndrome differentiation treatment of psoriasis vulgaris (PV) can achieve long-lasting effects, delay recurrence, and thus improve quality of life (Lu et al., 2012). In recent years, psoriasis has been mostly treated by syndrome depending TCM therapy from the view of blood in clinical practice in China. According to the epidemiological investigation of modern medicine and the consensus of TCM, the TCM syndromes in PV mainly include blood-stasis syndrome, blood-heat syndrome, and blood-dryness syndrome (Zhang et al., 2002; Zhang et al., 2009; He et al., 2014). On the basis of clinical epidemiological investigation, 2651 cases of psoriasis vulgaris were analyzed by investigation, multicenter, and large sample study. The TCM syndromes in psoriasis vulgaris mainly include the 3 types, and other syndromes were rarely seen, covering 0.6%. The concurrent syndromes mainly involve dampness, heat, blood stasis, and toxin (Zhang et al., 2008). The literature research, in total, 920 qualified articles from 1979 to 2010, also shows the distribution of Chinese medical syndrome in PV (Lu et al., 2010). The TCM evidence-based clinical practice guide for psoriasis vulgaris issued by the dermatology branch of Chinese Association of traditional Chinese medicine (2013) is also classified and defined in this way (Dermatology branch of Chinese Association of traditional Chinese Medicine, 2013). In the Guangdong Provincial Bureau of quality and technical supervision: psoriasis vulgar syndrome differentiation standard of traditional Chinese medicine, local standards of Guangdong Province (Guangdong Provincial Bureau of quality and technical supervision, 2018), shows that psoriasis is treated by blood, and the basic syndrome types are blood heat syndrome, blood stasis syndrome, and blood dryness syndrome. Two or three syndrome types can appear simultaneously in clinic, and other concurrent syndromes can be combined. The TCM syndrome diagnosis system of psoriasis vulgaris has been established, which can be used by clinicians for free (website: http://183.62.15.51:8912/index.html).

TCM syndrome differentiation diagnosis in the clinic is based on the comprehensive judgment considering many factors, such as the color and thickness of the skin lesion, the status of the tongue, the mood and mental state of the patient, and the whole metabolic state of the patient. It relies on the doctor’s clinical experience and subjective judgment. Therefore, it is necessary to study the objective basis of TCM syndrome differentiation in psoriasis. The development of omics techniques in systems biology provides powerful tools for understanding and interpretation of the TCM theory (Alessandro et al., 2012).

Metabonomics is a high-throughput omics technique, which is a new technique to analyze all low molecular weight metabolites in an organism or a cell qualitatively and quantitatively. Compared with other omics techniques, metabonomics reflects the final metabolic status of individuals more directly and comprehensively in response to pathophysiological stimuli or genetic modification (Nicholson et al., 1999). The systematic features of metabonomics are similar to the holistic view of TCM. Applying metabonomics approaches to material basis study of TCM syndrome differentiation is a beneficial attempt. Metabonomics technology research based on liquid chromatography coupled with mass spectrometry (LC-MS) and gas chromatography coupled with mass spectrometry (GC-MS) has been applied in the TCM syndrome differentiation studies and biomarker identification in coronary heart disease (Zhou et al., 2019) and rheumatoid arthritis (Gu et al., 2012). However, few studies were undertaken to explore the objective material basis of the TCM syndrome differentiation in patients with psoriasis. In this study, metabonomics strategy based on ultra-performance liquid chromatography coupled with quadruple-time-of-flight mass spectrometry (UPLC/Q-TOF-MS) was applied to investigate and compare the serum metabolic changes in patients with PV with different TCM syndrome types and healthy controls, as well as to discover the potential biomarkers to distinguish TCM syndromes.

## Materials and Methods

### Human Serum Collection

Outpatients (124) confirmed to have PV were recruited from the dermatology outpatient department of Guangdong Provincial Hospital of Chinese Medicine from April 2014 to July 2015. Healthy volunteers (45) were recruited from the physical examination department of Guangdong Provincial Hospital of Chinese Medicine. According to the diagnosis of TCM syndrome type, all patients with PV were divided into blood-heat type, blood-stasis type, and blood-dryness groups. The diagnostic criteria were based on the Western medicine diagnosis referring to the Guidelines of psoriasis treatment (2008), recommended by the American Academy of Dermatology. The TCM syndrome differentiation criteria referred to the judgment standard of TCM syndromes of psoriasis according to the “People’s Republic of China TCM Industry Standards” and “Principles of Clinical Research Guidelines for New Chinese Drugs.” Patients with PV with moderate to severe lesions (PASI > 10 or BSA > 10%) who agreed to sign informed consent were included. The study protocol (No. B2010-08-01) was approved by the institutional ethics committee of Guangdong Provincial Hospital of Chinese Medicine, and all the patients included in this study understood and signed informed consent.

Serum samples (124) of patients with PV (50 cases of the blood-stasis syndrome, 44 cases of the blood-dryness syndrome, 30 cases of the blood-heat syndrome) and 45 serum samples of healthy controls were collected and included in the study. Clinical characteristics of patients and controls are described in **Table 1**. Serum (4 mL) from all volunteers was collected on an empty stomach from the elbow vein blood in the morning, centrifuged, and extracted. The upper serum layer was stored at -80°C for further tests.

**Table 1:** Clinical characteristics of psoriasis vulgari patients with different TCM syndromes and healthy controls.

TCM Syndrome (Cases)	Male age (Average ± SD) (Cases)	Female age (Average ± SD) (Cases)
Blood-stasis(50)	42.0 ± 9.8 (30)	44.2 ± 10.9(20)
Blood-dryness(44)	42.9 ± 11.6 (26)	44.3 ± 13.6(18)
Blood-heat(30)	43.3 ± 9.4 (24)	51.2 ± 18.8 (6)
Healthy control(45)	40.1 ± 9.1 (24)	41.2 ± 8.1 (21)

### Reagents and Instruments

Acetonitrile was purchased from Merck (HPLC grade, Darmstadt, Germany). Formic acid was purchased from Fluka (Analytical grade, Buchs, Switzerland). Ultrapure water (18.2 MΩ) was prepared using a Milli-Q water purification system (Millipore, Bedford, MA, USA).

An ultra-performance liquid chromatography (Waters ACQUITY UPLC, USA) coupled with quadruple-time-of-flight mass spectrometry (AB SCIEX Triple TOF^™^ 5600, USA) was applied for serum analysis. Vortex mixer was purchased from Qilinbeier (VORTEX-5, Jiangsu, China), and the centrifuge was purchased from Beckman Coulter (AllegraTM X-22, Beckman Coulter Corporation, USA). Analyst^®^ TF (Version 1.5, AB SCIEX, USA), MarkerView™ (Version 1.2, AB SCIEX, USA), and Simca-P software (version 14.1, Umetrics, Umeå, Sweden) were applied.

### Sample Preparation

The frozen serum samples were placed in the 4°C refrigerator for freeze-thawing and vortexed for 2–5 seconds. Serum (150 μL) was transferred into a 1.5 mL Eppendorf tube and kept on ice, 600 μL of pure ice-cold acetonitrile was added into the tube and vortexed for 2 min, then centrifuged at 13,000 rpm for 20 min at 4°C. The whole supernatant was transferred into a 96-well collection plate for LC-MS analysis.

### LC-MS Analysis

Chromatographic separation was carried out using an ACQUITY BEH C18 analytic column (100× 2.1 mm, 1.7 µm, Waters); column temperature was set at 40°C. All metabolites were separated *via* linear gradient elution using water with 0.1% formic acid and acetonitrile with 0.1% formic acid as the mobile phase. The flow rate was 0.4 mL/min. The temperature of auto-sampler was set at 4°C and the sample injection volume was 2 μL.

The mass spectrometry parameters were as follows: MS scan range was set to m/z 100 to 1000 in positive electrospray ionization (ESI+) mode, ion source voltage flow (ISVF) was set to 5500, source temperature was set to 650°C, and collision energy (CE) was set to 10. The information-dependent acquisition function was enabled to obtain high resolution, high mass accuracy in the MS/MS spectra. All the acquired m/z data were real-time corrected using an independent reference to ensure higher accuracy.

### Raw Data Processing and Statistical Analysis

The raw spectrometric data were collected using the Analyst^®^ TF software. All of the raw data were imported to MarkerView™ software, and the metabolic profiling was processed for peak recognition and matching. Noise filtering, data normalization, and the peak list data were loaded to Simca-P software for multivariate statistical analysis. Unsupervised analysis (principal components analysis, PCA) and supervised analysis (partial least squares discriminate analysis, PLS-DA, and orthogonal signal correction partial least squares discriminate analysis, OPLS-DA) were applied to distinguish different TCM syndrome types of psoriasis.

### Identification of Potential Biomarkers

According to the value of variable importance in the projection (VIP) in the PLS-DA model, combined with t-test results, the potential candidate biomarkers were selected. Compounds were identified according to mass spectrometry isotope matching and the results of a database search in the human metabolome database (HMDB).

## Results

### Metabolic Profiling of Patients With PV With Different TCM Syndrome Types and Healthy Controls Was Established Using UPLC/Q-TOF-MS

**Figure 1:**
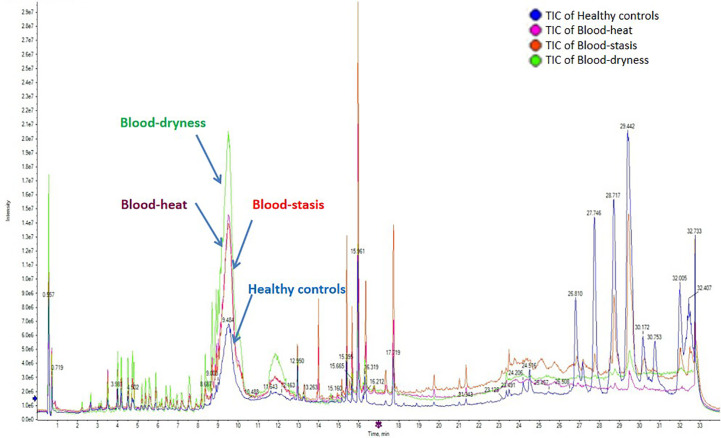
The total ion chromatograms (TIC) operating under positive ion mode. There were differences among the metabolic profilings of psoriasis vulgaris patients groups with blood-stasis, blood-heat, and blood-dryness and healthy control group.

UPLC/TOF-MS was applied to investigate the serum metabolic profiling of patients with psoriasis with different TCM syndrome types and healthy controls. The total ion chromatograms (TIC) operating under positive ion mode are shown in **Figure 1**. There were differences among the metabolic profiling of patients with PV with blood-stasis, blood-heat, and blood-dryness and the healthy control group. This suggested that there were a variety of metabolites in the serum related to the differentiation of TCM syndrome types in PV.

### Methodology Investigation

The sample pool was prepared by mixing 10 μL serum from each sample of all groups. According to the sample preparation method, a quality control (QC) sample was prepared for precision and stability investigation. One sample was injected six times, 10 peaks were selected from the TIC, and the retention time and ion response were stable. The relative standard deviation (RSD) of the peak intensity and the number of peaks of the six injections were less than 10%, indicating that the precision of the instrument was good and met the analysis requirements.

QC sample was prepared for stability test. A QC sample was set for injection every 10–15 samples, and all metabonomics samples were continuously injected. RSD of peak intensity and peak number of the six injections was less than 15%, indicating that the instrument and method were stable and reliable.

### The Multivariate Statistical Analysis Results for Patients With PV With Different TCM Syndrome Types

**Figure 2:**
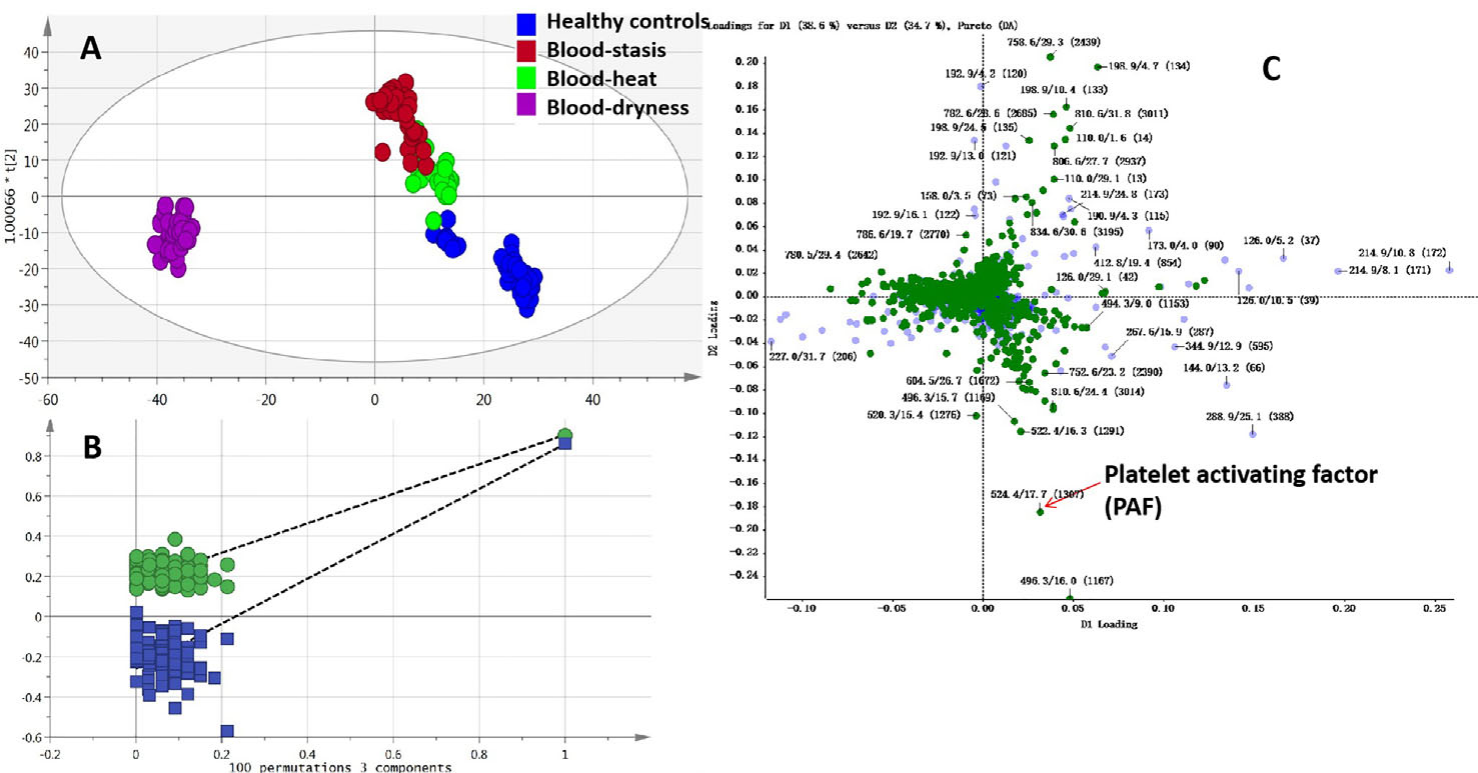
Multivariate data analysis and permutation test **(A)** OPLS-DA scores map, **(B)** Permutation test of OPLS-DA, **(C)** Loading map of PLS-DA.

The raw data of all groups were processed using MarkerView software for peak recognition and matching. Noise filtering, data normalization, and 3982 retention time_mass-to-charge ratio (RT_m/z) variables were screened. Isotope peaks were removed to avoid repeated calculation, and 2454 variables were loaded to Simca-P software for multivariate statistical analysis. PCA and PLS-DA, OPLS-DA were applied to study the clustering of samples of different syndrome types. The score map shows the correlation of samples in the coordinate map and the samples with similar metabolic status accumulated together. In contrast, different samples were distributed in different areas separately; therefore, the scores map could classify the experimental group samples according to metabolic differences. As shown in **Figure 2A**, blood-stasis group, blood-heat group, blood-dryness group, and healthy control group were clustered separately, indicating that there were significant metabolic differences among these groups. Loading map could reveal the components that contributed to the classification. The farther away from the origin, the larger the number of components that contributed to the classification. These components could be considered as potential biomarkers that interfere with normal physiological metabolism. In **Figure 2C**, the dots far away from the origin in the loading map were screened as the potential biomarkers. Permutation test with 100 iterations results showed that the predictive ability of this model was excellent. The model was validated as not over-fitting (**Figure 2B**).

### Potential Biomarker Screening and Identification

According to the PLS-DA pattern recognition results, the VIP values were sorted. Combined with the t-test, the significant difference (P < 0.05) of metabolites among the four groups were screened, and the candidate compounds from the loading map (**Figure 2C**) were identified. TOF-MS provided the precise molecular weight of the screened metabolites. The search for the compounds in the human metabolome database (HMDB), combined with isotopic molecular weight, mass spectrometry ionization fragmentation patterns, and biological significance, and the potential biomarkers were identified.

**Figure 3:**
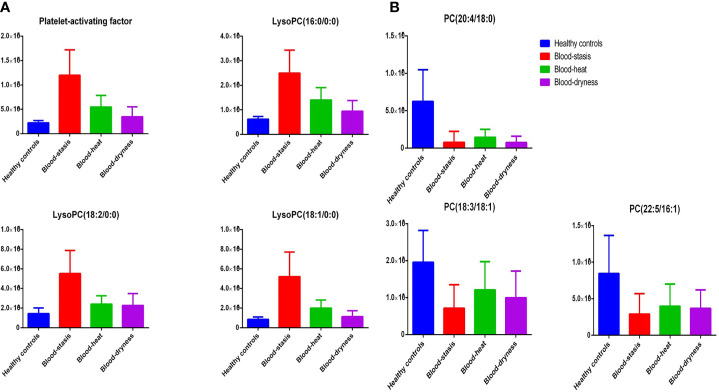
Typical metabolites with significant alterations. The peaks area of potential biomarkers in serum of the four groups were shown.

Fourteen significant metabolites in patients with psoriasis with different TCM syndromes and healthy controls were considered as potential biomarkers, as shown in **Table 2**. The compounds were mainly related to coagulation, mental function, inflammation, antibacterial action, and detoxification. Lipid compounds play an essential role in the development of psoriasis (Pietrzak et al., 2010; Pietrzak et al., 2019). In this study, all the psoriasis groups showed an abnormal lipid metabolism, which was characterized by the high level of lysophosphatidylcholine (LPC) (**Figure 3**) and low level of phosphatidylcholine (PC) (**Figure 3**) compared to the healthy control group.

**Table 2:** Potential biomarkers identification.

No.	m/z	Compound	Chemical Formula	Adducts	HMDB ID	Trend*
1	524.370	Platelet-activating factor	C26H54NO7P	M+H	HMDB0062195	↑
2	496.338	LysoPC(16:0)	C24H50NO7P	M+H	HMDB0010382	↑
3	520.337	LysoPC(18:2)	C26H50NO7P	M+H	HMDB0010386	↑
4	522.354	LysoPC(18:1)	C26H52NO7P	M+H	HMDB0002815	↑
5	550.385	LysoPC(20:1)	C28H56NO7P	M+H	HMDB10391	↑
6	544.340	LysoPC(20:4)	C28H50NO7P	M+H	HMDB10395	↑
7	835.528	PI(18:0/16:2)	C43H79O13P	M+H	HMDB09807	↑
8	613.393	Cholestane-3,7,12,25-tetrol-3-glucuronide	C33H56O10	M+H	HMDB10355	↑
9	782.570	PC(18:3/18:1)	C44H80NO8P	M+H	HMDB0008170	↓
10	806.571	PC(22:5/16:1)	C46H80NO8P	M+H	HMDB0008660	↓
11	810.601	PC(20:4/18:0)	C46H84NO8P	M+H	HMDB0008464	↓
13	806.561	Lactosylceramide (d18:1/12:0)	C42H79NO13	M+H	HMDB04866	↓
14	313.155	Phenylalanylphenylalanine	C18H20N2O3	M+H	HMDB13302	↓

*The trend was psoriasis groups compared to healthy control group.

Fourteen significant difference metabolites were screened out to be the potential biomarkers according to the value of variable importance for projection (VIP) value in PLS-DA model.

### Receiver Operating Characteristic Curve

**Figure 4:**
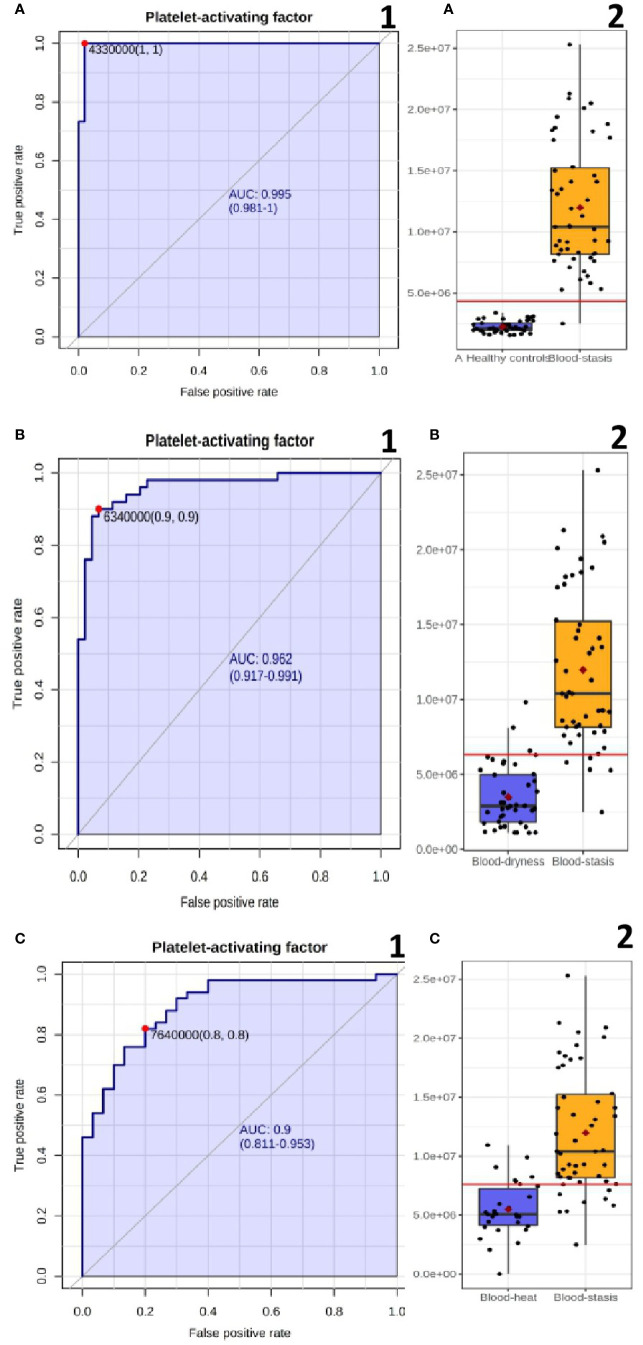
The ROC curve plots and boxplot of PAF **(A)** Blood-stasis vs Healthy controls; **(B)** Blood-stasis vs Blood dryness; **(C)** Blood-stasis vs Blood-heat.

One goal of metabonomic studies is biomarker discovery, which aims to identify a metabolite or a set of metabolites capable of classifying conditions or diseases with high sensitivity (true-positive rate) and specificity (true negative rate). ROC curve analysis is generally considered to be the gold standard for the assessment of biomarker performance. A compound was selected from the loading map (**Figure 2C**) and identified as the platelet-activating factor (PAF). PAF was evaluated using a classical ROC curve analysis. **Figure 4** shows the results of the ROC curve of PAF in the blood-stasis group. Compared to the healthy control group, the area under the curve (AUC) was 0.995 (**Figure 4A-1**), and the boxplot of two groups is shown in **Figure 4A-2**. Compared to the blood-dryness group, AUC was 0.962 (**Figure 4B-1**), and the boxplot of the two groups is shown in **Figure 4B-2**. Compared to the blood-heat group, AUC was 0.9 (**Figure 4C-1**), and the boxplot of the two groups is shown in **Figure 4C-2**. All AUC values were higher than 0.9, suggesting that PAF had good predictive ability in distinguishing the blood-stasis group from the other groups. PAF might be a valuable biomarker in patients with psoriasis with blood-stasis syndrome.

## Discussion

There were 14 potential biomarkers in **Table 2**, mainly involved in glycerophospholipid metabolism pathway, also related to linoleic acid metabolism, sphingolipid metabolism, arachidonic acid metabolism, and pentose and glucuronate interconversions. In recent years, lipid metabolism abnormalities have been reported in patients with psoriasis (Taheri et al., 2014). Psoriasis patients are prone to diabetes, hyperlipidemia, hypertension, and other metabolic diseases (Ribeiro et al., 2014), suggesting that psoriasis has a close relationship with metabolic syndromes, involving phospholipid, fatty acid, amino acid, arachidonic acid metabolisms, and other metabolic cycles.

In this study, all psoriasis groups showed an abnormal lipid metabolism, which was characterized by the high level of LPC and low level of PC compared to the healthy control group. The level of LysoPC(16:0), LysoPC(18:2), LysoPC(18:1), and LysoPC(20:1) in the serum of patients with psoriasis were significantly higher than that of healthy controls, whereas the level of PC(18:3/18:1), PC(22:5/16:1), and PC(20:4/18:0) were significantly higher than that of the healthy controls. The result was consistent with the previous lipidomics research on psoriasis (Zeng et al., 2017). LPC is the main active component of oxidized LDL, which has a wide range of biological effects, including the development of atherosclerosis. LPC is a degradation product of PC. It can induce inflammatory response, increase oxidative stress, interfere with vascular endothelial function, and destroy plaque stability. LPC plays an essential role in the prevention, diagnosis, and treatment of atherosclerosis. Brown adipose tissue (BAT) recently emerged as a potential therapeutic target in the treatment of obesity and associated disorders due to its fat-burning capacity. The concentrations of lysophosphatidylcholine-acyl (LysoPC-acyl) C16:1, LysoPC-acyl C16:0, and phosphatidylcholine-diacyl C32:1 show strong positive correlations with BAT volume as well as BAT activity (Boon et al., 2017).

PAF, also known as PC(O-16:0/2:0), is a ubiquitous, potent phospholipid activator and mediator of inflammation that has an important role in the pathogenesis of inflammatory disorders and cardiovascular disease. PAF causes platelet aggregation and anaphylaxis. PAF is synthesized continuously in low quantities in many different types of cells, especially those involved in host defense, such as macrophages, monocytes, granulocytes, neutrophils, platelets, and endothelial cells. Platelet-activating factor receptor is a G-protein coupled receptor located on the cell membranes of a variety of cells. Once bound to its receptor, PAF mobilizes calcium and activates a wide range of signaling pathways. In our study, the PAF level of blood-stasis syndrome was significantly higher than that of non blood-stasis syndrome, and that of non blood-stasis syndrome was significantly higher than that of healthy controls (**Supplement Table 1**), and the ROC curve showed excellent predictive ability (**Figure 4A**). The relationship of PAF and psoriasis had been studied by several researchers previously (Judge et al., 1994; Lzaki et al., 1996; Greaves and Judge, 1997), but there were different comments about it. In recent years, it has been increasingly recognized that platelet activation seems to be a major pathogenic factor in psoriasis (Elbers et al., 2010), and platelet activation indexes generally suggest abnormal platelet function and are given an additional role in the diagnosis of psoriasis. Platelets are also immune cells that initiate and regulate immune and inflammatory processes, except as the principal mediator of hemostasis and thrombosis, and platelet dysfunction is deeply involved in the pathogenesis of psoriasis (Fan et al., 2020).The platelet activating factor has been reported in coronary heart disease with blood-stasis syndrome (Zheng et al., 2012) and primary dysmenorrhea with blood-stasis tongue figure (Yang and Chen, 2011), but there is no report in psoriasis with blood-stasis. PAF cannot be a characteristic diagnostic marker of psoriasis disease, but it is expected to be used as potential biomarkers for diagnosis and differentiation of blood-stasis syndrome in PV, and the increase of platelet-activating factor may be a pathogenesis of blood-stasis syndrome.

## Conclusion

We developed an untargeted metabonomics method to investigate the differences among patients with psoriasis with different TCM syndromes and healthy controls. Fourteen potential biomarkers were screened and identified. Lower level of phosphatidylcholine (PC) and higher level of lysophosphatidylcholine (LPC) were shown in psoriasis patients. We propose PAF as a biomarker for diagnosis and differentiation of blood-stasis syndrome, levels of which show that there is an objective material basis in TCM syndrome differentiation of psoriasis.

## Data Availability Statement

The raw data supporting the conclusions of this article will be made available by the authors, without undue reservation.

## Ethics Statement

The studies involving human participants were reviewed and approved by institutional ethics committee of Guangdong provincial hospital of Chinese medicine. The patients/participants provided their written informed consent to participate in this study. Written informed consent was obtained from the individual(s) for the publication of any potentially identifiable images or data included in this article.

## Author Contributions

C-jL and LH supervised the whole experiments. LL designed this study and performed the practical work and completed the experiments. D-nY, J-wD, Y-hY, and HD diagnosed patients and collected samples. YL and J-aW provided help during experiments. All authors contributed to the article and approved the submitted version.

## Funding

This work was supported by natural science foundation of China (81302736), Guangdong natural science foundation (S2011040005849), Guangdong Province Science and Technology Planning Project (2017A050506041, 2017B030314166, 2019A1515010636, 2020B1111100006, 2020A1515010607), Guangdong Provincial Department of Education Project (2018KQNCX046), Guangzhou Science and Technology Project (201807010051), and Guangdong Provincial Hospital of Chinese Medicine Special Fund (YN2018HK01, YN2018ZD08, YN2018RBA02, YN2016XP02, YN2019QJ04, YN2019QJ08, 2019KT1313).

## Conflict of Interest

The authors declare that the research was conducted in the absence of any commercial or financial relationships that could be construed as a potential conflict of interest.

## References

[B1] Alessandro B. Maria L. Garcia B. Enrica B. Xu Q. H. Li H. G. (2012). Omic techniques in systems biology approaches to traditional Chinese medicine research: Present and future. J. Ethnopharmacol. 140, 535–544. 10.1016/j.jep.2012.01.055 22342380

[B2] Boon M. R. Bakker L. E. H. Prehn C. Adamski J. Vosselmanet M. J. Jazet I. M. (2017). LysoPC-acyl C16:0 is associated with brown adipose tissue activity in men. Metabolomics 13, 48. 10.1007/s11306-017-1185-z 28316560 PMC5334436

[B3] Dermatology branch of Chinese Association of traditional Chinese Medicine (2013). Evidence based clinical practice guide of traditional Chinese medicine for psoriasis vulgariVersion). J. Tradit. Chin. Med. 55, 76–82. 10.13288/j.11-2166/r.2014.01.021

[B4] Elbers M. E. Gerritsen M. J. Kerkhof P. C. (2010). The effect of topical application of the platelet-activating factor-antagonist, Ro 24-0238, in psoriasis vulgaris–a clinical and immunohistochemical study. Clin. Exp. Dermatol. 19 (6), 453–457. 10.1111/j.1365-2230.1994.tb01246.x 7889665

[B5] Fan Z. Wang L. Jiang H. Yong L. Wang Z. C. (2020). Platelet Dysfunction and Its Role in the Pathogenesis of Psoriasis. Dermatology 1–10. 10.1159/000505536 32349003

[B6] Greaves M. W. Judge M. R. (1997). Platelet activating factor in psoriasis. Br. J. Dermatol. 136 (3), 467. 10.1111/j.1365-2133.1997.tb14968.x 9115938

[B7] Gu Y. Lu C. Zha Q. Kong H. Lu X. Lu A. (2012). Plasma metabonomics study of rheumatoid arthritis and its Chinese medicine subtypes by using liquid chromatography and gas chromatography coupled with mass spectrometry. Mol. Biosyst. 8, 1535–1543. 10.1039/c2mb25022e 22419152

[B8] Guangdong Provincial Bureau of quality and technical supervision (2018). “Psoriasis vulgar syndrome differentiation standard of traditional Chinese medicine,” (Local standards of Guangdong Province: Guangdong Provincial Bureau of quality and technical supervision), DB44/T 2120–2018.

[B9] He Z. H. Wang D. M. Lu C. J. Ou A. H. (2014). The Distribution of TCM Syndromes of Vulgaris Psoriasis:Correspondence Analysis of the Relationships Between TCM Syndromes and Disease Stages. Chin. J. Dermatovenereol. 28, 22–25. 10.13735/j.cjdv.1001-7089.2014.0022

[B10] Judge M. R. Barr R. M. Mallet A. I. Courtney F. Black A. K. Greaves M. W. (1994). Platelet activating factor (PAF) and lyso-PAF in psoriasis[J]. Arch. Dermatol. Res. 286 (7), 376–379. 10.1007/BF00371796 7818279

[B11] Lu C. J. Zeng Z. Xie X. L. Ning J. (2010). Distribution of Chinese Medical Syndrome in Ordinary Psoriasis: Literature from 1979 to 2010. J. Tradit. Chin. Med. 53, 959–961.

[B12] Lu C. J. Yu J. J. Deng J. W. (2012). Disease-syndrome combination clinical study of psoriasis: Present status, advantages, and prospects. Chin. J. Integr. Med. 18, 166–171. 10.1007/s11655-012-1006-1 22466939

[B13] Lzaki S. Yamamoto T. Goto Y. Lshimaru S. Matsuzakiet M. (1996). Platelet-activating factor and arachidonic acid metabolites in psoriatic inflammation. Br. J. Dermatol. 134 (6), 1060–1064. 10.1111/j.1365-2230.1994.tb01246.x 8763425

[B14] Nestle F. O. Kaplan D. H. Barker J. (2009). Psoriasis. N. Engl. J. Med. 361, 496–509. 10.1056/NEJMra0804595 19641206

[B15] Nicholson J. K. Lindon J. C. Holmes E. (1999). ‘Metabonomics’: understanding the metabolic responses of living systems to pathophysiological stimuli via multivariate statistical analysis of biological NMR spectroscopic data. Xenobiotica 29, 1181–1189. 10.1080/004982599238047 10598751

[B16] Pietrzak A. Chodorowska G. Szepietowski J. Zalewska-Janowska A. Krasowska D. Hercogová J. (2010). Psoriasis and serum lipid abnormalities. Dermatol. Ther. 23, 160–173. 10.1111/j.1529-8019.2010.01311.x 20415824

[B17] Pietrzak A. Chabros P. Grywalska E. Kicinski P. Franciszkiewicz-Pietrzak K. Krasowska D. (2019). Serum lipid metabolism in psoriasis and psoriatic arthritis - An update. Arch. Med. Sci. 15, 369–375. 10.5114/aoms.2018.74021 30899289 PMC6425200

[B18] Ribeiro B. Bittencourt F. V. Gontijo B. Andrade G. (2014). Comorbidities and cardiovascular risk factors in patients with psoriasis. Anais Brasileiros Dermatol. 89, 735–744. 10.1590/abd1806-4841.20142874 PMC4155951 25184912

[B19] Taheri S. M. Hedayati M. T. Shokohi T. Haj H. Z. (2014). Serum lipids and lipoproteins in patients with psoriasis. Arch. Iranian Med. 17, 343–346. 0141705/AIM.007 24784863

[B20] Yang A. P. Chen Q. (2011). Correlation between blood-stasis tongue figure and platelet activating factor (PAF) and acetyl hydrolase of PAF (PAF-AH) in patients with primary dysmenorrhea. Zhongguo Zhong XI Yi Jie He Za Zhi 31 (3), 331–333. 10.1097/MOP.0b013e328341d1da 21485072

[B21] Zeng C. Wen B. Hou G. Lei L. Mei Z. Jia X. (2017). Lipidomics profiling reveals the role of glycerophospholipid metabolism in psoriasis. Gigascience 6, 1–11. 10.1093/gigascience/gix087 PMC5647792 29046044

[B22] Zhang G. Wang P. Wang J. Jiang C. Deng B. Li P. (2008). Study on the distribution and development rules of TCM syndromes of 2651 psoriasis vulgaris cases. J. Tradit. Chin. Med. 29, 894–896. 10.1016/s0254-6272(09)60064-9 19894384

[B23] Zhang G. Z. Wang J. S. Wang P. Jiang C. Y. Deng B. X. Li P. (2009). Distribution and Development of the TCM Syndromes in Psoriasis Vulgaris. J. Tradit. Chin. Med. 29, 195–200. 10.1016/S0254-6272(09)60064-9 19894384

[B24] Zhang B. Z. (2002). Preliminary study on essence of TCM syndrome typing of psoriasis and its objective basis. CJIM 8, 254–255. 10.1007/BF02934397

[B25] Zheng G. H. Xiong S. Q. Mei L. J. Chen H. Y. Wang T. Chu J. F. (2012). Elevated Plasma Platelet Activating Factor, Platelet Activating Factor Acetylhydrolase Levels and Risk of Coronary Heart Disease or Blood Stasis Syndrome of Coronary Heart Disease in Chinese: A Case Control Study[J]. Inflammation 35 (4), 1419–1428. 10.1007/s10753-012-9455-4 22430230

[B26] Zhou H. Li L. Zhao H. Wang Y. Du J. Peng J. (2019). A Large-Scale, Multi-Center Urine Biomarkers Identification of Coronary Heart Disease in TCM Syndrome Differentiation. J. Proteome Res. 18, 1994–2003. 10.1021/acs.jproteome.8b00799 30907085

